# COVID-19 and Health Systems Functioning in Sub-Saharan Africa Using the “WHO Building Blocks”: The Challenges and Responses

**DOI:** 10.3389/fpubh.2022.856397

**Published:** 2022-04-04

**Authors:** Hubert Amu, Robert Kokou Dowou, Farrukh Ishaque Saah, John Adebayo Efunwole, Luchuo Engelbert Bain, Elvis Enowbeyang Tarkang

**Affiliations:** ^1^Department of Population and Behavioural Sciences, School of Public Health, University of Health and Allied Sciences, Hohoe, Ghana; ^2^Department of Epidemiology and Biostatistics, School of Public Health, University of Health and Allied Sciences, Hohoe, Ghana; ^3^Lincoln International Institute for Rural Health, College of Social Science, University of Lincoln, Lincoln, United Kingdom

**Keywords:** COVID-19, health systems, sub-Saharan Africa (SSA), WHO building blocks, perspective

## Abstract

Sub-Saharan Africa (SSA) has made major progress in improving access to health care over the past three decades. Despite efforts made toward achieving universal health coverage, the health systems of countries in the sub-region are inundated by a myriad of challenges that have become more virulent amid the COVID-19 pandemic. This paper discusses the health systems challenges and responses in SSA amidst the COVID-19 using the World Health Organization's (WHO) building blocks of health systems functioning. Long-lasting abysmal health system financing and insufficient government investment in SSA pose major challenges to the effective health systems functioning amid the COVID-19 pandemic. This situation also makes it difficult for the health system to meet the demands of the COVID-19 pandemic and at the same time, cater for other essential health services. Countries in SSA must prioritize the reformation of their health systems through effective health system policy development and implementation, human resources development, training, service delivery, governance and regulation, and sustainable health financing.

## Introduction

**Figure 1:**
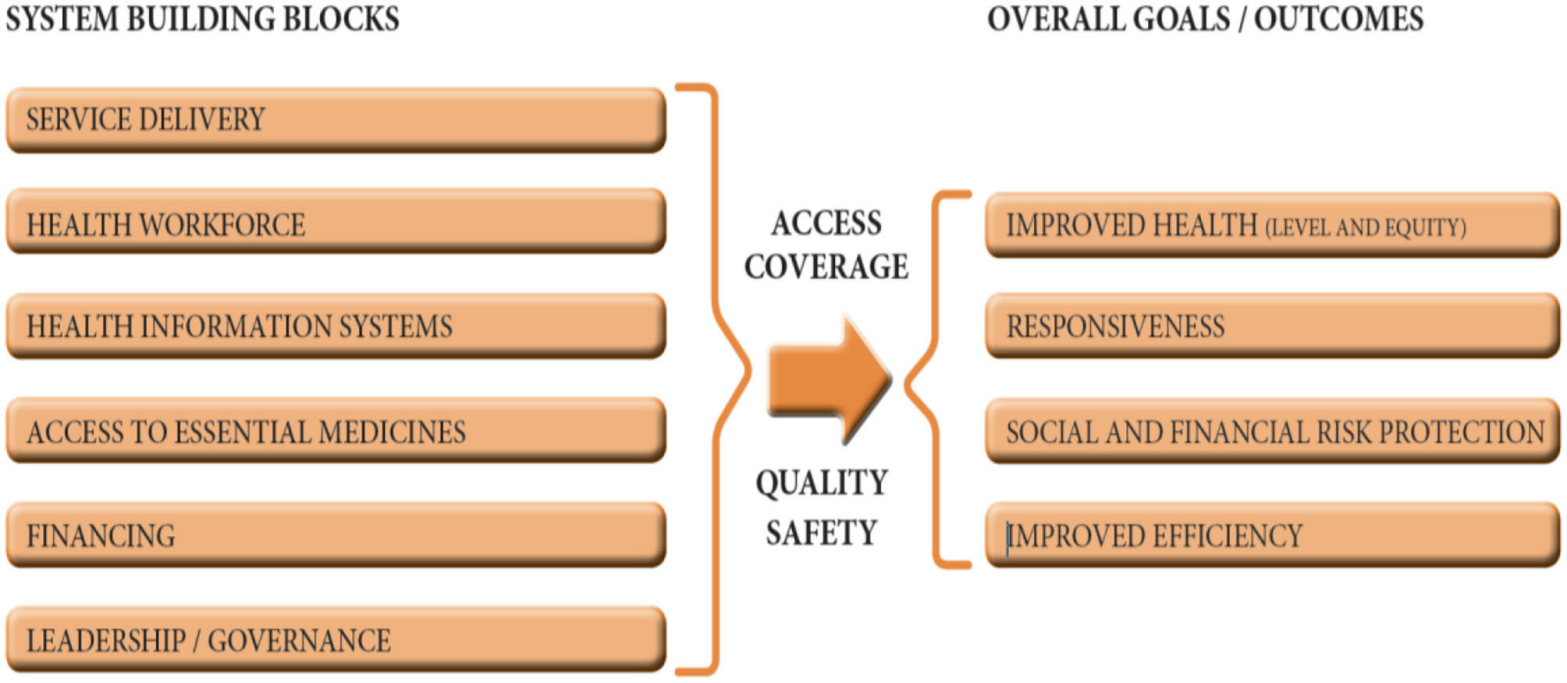
The six building blocks of a health system: aims and desirable attributes. Source: World Health Organization (6).

In December 2019, a novel coronavirus (SARS-CoV-2) which causes the coronavirus disease 2019 (COVID-19) was isolated in China (1). The WHO on March 11, 2020, declared COVID-19 as a global pandemic after a rapid spread of the virus (2) which has caused a worldwide disruption to health systems. Over the past 2 years that the virus broke out, about 386,548,962 confirmed cases including 5,705,754 deaths have been recorded as of 2nd February, 2022 (3). At the same time the world is making all efforts to reduce the burden and mortality of the pandemic through vaccination with a total of 10,040,768,270 vaccine doses being administered (3). Health systems constitute the foundation for achieving the third sustainable development goal (SDG) of ensuring health for all at all ages by the year 2030 (4). A health system refers to all activities whose primary purpose is to promote, restore, and maintain health (5). The primary aim of every health system is to protect and improve the health of the people, hence it is concerned with people's health (6). Despite efforts made over the years toward achieving health for all, the health systems of SSA countries are undermined by a myriad of challenges which have been exacerbated by the COVID-19 pandemic. Key challenges in the health system in SSA include inadequate human resources, insufficient financing through low budgetary allocation, inadequate availability of essential medicines, and poor leadership and management (7). This paper discusses the challenges and responses in strengthening health systems in SSA in the midst of the COVID-19 using the WHO building blocks of health systems functioning. The building blocks constitute the WHO framework that describes health systems in terms of six core components comprising service delivery, health workforce, health information systems (HIS), access to essential medicines, financing, and leadership/governance (6) (Figure 1).

Using the health systems building blocks, the key research question of this paper is: What are the delivery, health workforce, health information systems (HIS), access to essential medicines, financing, and leadership/governance challenges and responses toward ending the COVID-19 pandemic in sub-Saharan Africa?

## Service Delivery

Effective health service delivery is central to the achievement of the health-related Sustainable Development Goals (SDGs), which include the delivery of interventions to reduce child mortality, maternal mortality and the burden of HIV/AIDS, tuberculosis and malaria by the end of 2030 (4). In SSA, healthcare is mostly provided at five functional levels. These are Community (health posts), sub-district (health centers and clinics), District (District Hospitals/health directorates), Regional (Regional hospitals/health directorates) and national (tertiary/quaternary hospitals). Health service delivery is the immediate output of the inputs into the health system, such as the health workforce, procurement and supplies, financing and governance (6). COVID-19 has exposed the deficit in health services prevailing in SSA. The inadequacy of laboratories and testing kits in most SSA countries to carry out mass laboratory testing of samples collected from suspected COVID-19 cases has resulted in delays in testing. In most cases, it takes more than the recommended 2–3 days to receive results. Also, the limited number of beds and other facilities including lack of isolation centers has led to the premature discharge of COVID-19 patients to go self-isolate in their various homes while no specific strategy is put in place to follow them up (8).

There have been some successful responses across the sub-region toward improving healthcare access in terms of the availability of health facilities. Many governments are investing in building more health facilities and specialized centers. For instance, the Ghana government with funds from the private sector put up the first infectious disease isolation and treatment center; Ghana Infectious Disease Centre (9). This is consistent with the suggestion by Gebremeskel et al. (10) that private sector involvement in health system financing and administration has the potential of resolving existing infrastructural limitations. Again, the government has initiated processes to construct 111 District and Regional level health facilities to bridge the accessibility gap (11). Similar infrastructural improvement efforts such as building hospitals and installing or building oxygen plants are ongoing in countries like Nigeria, Kenya, and Tanzania (12–14).

## Health Workforce

The capacity of a nation to deliver adequate health services to its population depends principally on the skills, competence, knowledge, dedication, motivation and deployment of well-trained health professionals (6). However, many countries in SSA lack the adequate human resources (number, distribution, skills-mix) required to deliver essential health services. The inadequacy of human resources in SSA is more exposed since the sub-district recorded its first COVID-19 case. The existing shortage of qualified health professionals to deliver essential services to patients during the pandemic has resulted in the negligence of many essential services such as child and maternal health service delivery, chronic non-communicable diseases (CNCDs), and prevention and treatment of infectious diseases (15). This negligence could be attributed to the fact that the limited health professionals are redeployed into the provision of COVID-19 management services including contact tracing, triaging, laboratory testing and case management services.

There have been some successful responses as well. COVID-19 has, for instance, contributed to improving the skills of health professionals as it has provided opportunities for training and orientation of health professionals in different health service competencies including infection control and prevention protocol, laboratory testing guideline and management of infectious diseases like COVID-19. Regarding the laboratory testing services, the pandemic has contributed to improvements in health technology in SSA. For instance, in Ghana, to deal with lack of laboratory equipment and supplies that delay the COVID-19 testing, Kwame Nkrumah University of Science and Technology (KNUST) and Incas Diagnostics has fast-tracked the urgent development of a rapid diagnostic test kit that is approved by Food and Drug Authority (FDA) of the country for emergency use (16). A similar innovative response is observed in Senegal, which developed its first-ever 3D-printed ventilators and COVID-19 testing kit by Institute Pasteur (17).

## Health Information Systems

A health information system constitutes a system designed to manage healthcare data. The HIS include collection, storing, managing (analysis and synthesis), transmitting, and converting the data into information and use of clients' medical records for decision making (18). Complete and reliable information is essential for health priority setting and decision-making across all health systems. The data-based health-related decision making underpins the effectiveness of the health system's (hospital) operational management (6). HIS are the foundations of public health in every country especially during a pandemic like COVID-19 (18). Despite the critical role data play in public health decision-making during health emergencies (COVID-19), SSA countries are known to have deficient health information management systems (19). There are deep discrepancies regarding the COVID-19 “actual” cases reported by the health authorities in SSA. Official COVID-19 statistics, for instance, do not accurately reflect the true cases and deaths reported (20).

Despite the inadequate HIS in SSA even before the pandemic, it is important to indicate some successful responses experienced by SSA regarding the HIS. Some countries in SSA have, for instance, deployed diverse digital information system infrastructures and innovation to increase pandemic communication (daily case updates), contact tracing, tacking vaccination coverage, to effectively manage the COVID-19 and keep the general population informed (21, 22). To respond effectively to the population's demand for regular and timely information on the pandemic, Nigeria, Ethiopia, and South Africa developed a data-driven health information portal with a rapid response component using call centers (21). The response of the public to the call centers in the respective countries was overwhelming. For instance, in South Africa, call center of country's National Public Health Institute (NPHI), the National Institute for Communicable Diseases, received about 146,000 calls in 1 day (23). Similarly, to be able to effectively trace suspected contacts of confirmed cases and prevent further spread of the virus, Ethiopia and Ghana have designed several mobile Apps (COVID-19 tracker mobile app) that trace contact, share data and patient information among the health workers for timely response (24).

## Health Financing and Access to Essential Medicines

Access to essential health intervention including essential medicines and supplies was already limited in SSA before COVID-19 (22). Some SSA nations experience poor availability of essential medicines in health facilities, frequent stock-outs, substandard treatments, and suboptimal prescription and use of medicines (25). The lack of financial power of SSA countries to invest in health technology, storage facilities for pharmaceutical products and improvements in procurement practices, prove how incapable they are in developing vaccines to fight COVID-19. As such, they become completely dependent on foreign aid for the vaccines as has always been the situation. The persistent health financing constraints in SSA made it difficult for the countries to procure essential medicines to manage COVID-19 cases.

Health financing is fundamental to the effective functioning of health care systems leading to the achievement of the SDGs, including universal health coverage by 2030 (26). Health financing systems in SSA are largely characterized by high out-of-pocket payment, high dependence on external (donor) funding, low government spending and under-developed insurance schemes (21, 26). In 2017, out of pocket health spending was estimated to have exceeded 70% of current health expenditure in Cameroon, Equatorial Guinea, Nigeria and Sudan (27). For instance, foreign funding (donor) of the health system accounts for more than 60% of health expenditure in Mozambique and Malawi (28).

Responding to the inadequacy of financial risk protection in SSA, countries including Ghana, Tanzania, Nigeria, Ethiopia, Kenya, and Rwanda have formulated and implemented national health insurance schemes (29, 30). Despite these efforts, the majority of the population in SSA still suffer financial barriers as out-of-pocket expenditure is required before essential medical care can be delivered, even in emergencies. In such situations, the most vulnerable (poor), therefore, bear the highest burden of diseases and high levels of health expenditure. The already fragile health systems are overburdened with the grave task to address the COVID-19 pandemic. The response to the pandemic which requires much financial investment from the nations has resulted in low budget allocation to essential health services delivery. This has led to high prevalence rates and preventable death from the pandemic.

Despite the shortfall of the health financing and leadership in SSA during the COVID-19 pandemic, it is also worth pointing out some successful responses that are observed during the pandemic. To effectively mobilize the needed resources to manage the pandemic (public health and clinical care response), diverse resource mobilization was explored, including special COVID-19 response taxes, international grants, cooperate organization donations, political parties fundraising and private citizen donations (31). In Nigeria for instance, the private sector organizations through a coalition known as Coalition against COVID-19 (CACOVID) and Dangote, contributed over US$55.7 million and US$5.1 million, respectively, to COVID-19 response (32).

## Leadership/Governance

Poor governance and financial challenges are linked to ineffective integration and delivery of adequate health services in SSA (12). The leadership and management challenges include lack of political will, corruption in health systems, poor resource management. The endemic poor governance in SSA leads to weak institutions and ineffective implementation of health policies, increased healthcare costs, lack of availability and accessibility to health services, reduced efficiency and effectiveness, dissatisfaction among health professionals due to poor motivation, and ultimately poor health outcomes for the population (33). Strong and corruption-free governance is crucial for a robust health system and resilient health system in SSA. However, resources that are mobilized internally by citizens themselves or externally to fight COVID-19 are either mismanaged or diverted to other areas by leadership, leading to inadequate provision of needed medical logistics and supplies such as PPE.

Some governments across SSA have demonstrated political leadership during COVID-19 by establishing several fiscal policies to improve funding of their health systems. These innovative approaches include effective collection of corporate and business taxes and swopping debt reduction for domestic investment in health systems (32). For example, to effectively respond to the pandemic and its economic effects, the government of Nigeria approved US$2.3 million as its fiscal stimulus package. The Government of Ghana also GHc323 million as relief for frontline health workers (32).

## Conclusion

Long-lasting abysmal health system financing and insufficient government investment in SSA pose major challenges to the effective health service provision amid the COVID-19 pandemic. This situation also makes it difficult for the health system to meet the demands of the COVID-19 pandemic and at the same time, cater for essential services. Despite the constraints faced, interventions introduced by leaders of the various countries induced some resilience among the populace. Countries in SSA must prioritize the reformation of their health systems through effective health system policy development and implementation, human resources development, training, service delivery, governance and regulation, and sustainable health financing. Future studies could also examine the effectiveness of the responses to COVID-19 in sub-Saharan Africa.

## Data Availability Statement

The original contributions presented in the study are included in the article/supplementary material, further inquiries can be directed to the corresponding author/s.

## Author Contributions

HA conceived the study. HA, RKD, FIS, and JAE wrote the initial draft of the manuscript. LEB and EET provided critical comments which improved the scientific quality of the manuscript. All authors contributed to a review of the initial manuscript draft, gave consent, and approved the final draft of the manuscript for submission.

## Conflict of Interest

The authors declare that the research was conducted in the absence of any commercial or financial relationships that could be construed as a potential conflict of interest.

## Publisher's Note

All claims expressed in this article are solely those of the authors and do not necessarily represent those of their affiliated organizations, or those of the publisher, the editors and the reviewers. Any product that may be evaluated in this article, or claim that may be made by its manufacturer, is not guaranteed or endorsed by the publisher.
